# Project-based learning in remote teaching for undergraduate nursing students

**DOI:** 10.1590/1980-220X-REEUSP-2022-0058en

**Published:** 2022-08-22

**Authors:** Daniela Miori Pascon, Débora Rodrigues Vaz, Heloísa Helena Ciqueto Peres, Valéria Marli Leonello

**Affiliations:** 1 Universidade de São Paulo, Escola de Enfermagem, Departamento de Orientação Profissional, São Paulo, SP, Brazil.

**Keywords:** Universities, Education, Nursing, Education, Distance, Problem-Based Learning, Students, Nursing, Universidades, Educación en Enfermería, Educación a Distancia, Aprendizaje Basado en Problemas, Estudiantes de Enfermería, Educação Superior, Educação em enfermagem, Aprendizagem on-line, Aprendizagem ativa, Aprendizagem Baseada em Projetos, Estudantes de enfermagem

## Abstract

**Objective:**

To report the experience of using the Project-Based Learning methodology, in emergency remote teaching, with undergraduate nursing students.

**Method:**

The study was carried out in the course “Educational Actions in Nursing Practice”, developed remotely in 2020, during the pandemic caused by the new coronavirus, in the Bachelor’s and Licanciate Degrees in Nursing at a public university in the state of São Paulo. The course used Project-Based Learning through the following phases: anchoring; driving question; investigation and research; creation and development; and presentation of the results as an active learning methodology, with the formation of small groups of students and tutors and process evaluation.

**Results:**

The students developed educational projects in health through a virtual learning environment, platforms, and digital tools.

**Conclusion:**

The methodology adopted and the use of digital technologies allowed the achievement of the proposed objectives, the motivation and autonomy of the students throughout the remote teaching process, and the development of competences for the elaboration of projects in health education for nursing training.

## RESUMO

**Objetivo:**

Relatar a experiência do uso da metodologia de Aprendizagem Baseada em Projetos, no ensino remoto emergencial, com estudantes ingressantes da graduação em enfermagem.

**Método:**

O estudo foi realizado na disciplina de “Ações educativas na prática de enfermagem”, desenvolvida de maneira remota no ano de 2020, durante o período da pandemia causada pelo novo coronavírus, no Curso de Bacharelado e Licenciatura em Enfermagem de uma universidade pública do estado de São Paulo. A disciplina utilizou a Aprendizagem Baseada em Projetos por meio das fases: âncora; questão motriz; investigação e pesquisa; criação e desenvolvimento; e apresentação dos resultados, como metodologia ativa de aprendizagem, com a formação de pequenos grupos de estudantes e tutores e avaliação processual.

**Resultados:**

Os estudantes desenvolveram projetos educativos em saúde por meio de ambiente virtual de aprendizagem, plataformas e ferramentas digitais.

**Conclusão:**

A metodologia adotada e a utilização das tecnologias digitais permitiram alcançar os objetivos propostos, a motivação e a autonomia dos estudantes durante todo o processo de ensino remoto, e o desenvolvimento de competências para a elaboração de projetos no ensino em saúde na formação do enfermeiro.

## RESUMEN

**Objetivo:**

Relatar la experiencia del uso de la metodología de Aprendizaje Basado en Proyectos, en la enseñanza a distancia de emergencia, con estudiantes de enfermería.

**Método:**

El estudio fue realizado en la disciplina de “Acciones Educativas en la Práctica de Enfermería”, desarrollada remotamente en 2020, durante el período de la pandemia provocada por el nuevo coronavirus, en el Curso de Bachillerato y Licenciatura en Enfermería de una universidad pública. La disciplina utilizó el Aprendizaje Basado en Proyectos a través de las fases: ancla; pregunta orientadora; investigación; creación y desarrollo; y presentación de los resultados, como metodología de aprendizaje activo, con formación de pequeños grupos de alumnos y tutores y evaluación del proceso.

**Resultados:**

Los estudiantes desarrollaron proyectos educativos en salud a través de un entorno virtual de aprendizaje, plataformas y herramientas digitales.

**Conclusión:**

La metodología adoptada y el uso de tecnologías digitales posibilitaron el logro de los objetivos propuestos, la motivación y autonomía de los estudiantes a lo largo del proceso de enseñanza remota, y el desarrollo de habilidades para la elaboración de proyectos en educación para la salud en la formación de enfermeros.

## INTRODUCTION

The World Health Organization (WHO), in March 2020, declared COVID-19, caused by SARS-CoV2, a pandemic^(1)^. The scope of the spread of the virus around the world has made countries adopt contagion reduction strategies^(2)^, including social isolation, which directly affected education systems with the widespread closure of educational institutions such as schools, colleges, and universities^(3,4)^.

The suspension of in-person activities led Higher Education Institutions (IESs) to quickly adapt, in an improvised way, their teaching strategies and tools, characterizing Emergency Remote Teaching (*ERE*).

The *IES*s adopting *ERE* faced several challenges, including the unequal access to the internet and electronic devices by students and the lack of technological support and training for teachers to carry out the planning and development of activities in the remote format. Moreover, the continuity of activities challenged professors to rethink the methodologies, such as them the active methodologies, previously adopted in the in-person format, to remote teaching^(4,5)^.

Adopting active methodologies presupposes that the professors involved, based on pedagogical mediation, stimulate the protagonism and autonomy of students in the individual and collaborative learning process^(6)^. Developing such premises in the remote environment is an opportunity to rethink the teaching process^(4)^.

In nursing education, particularly in the disciplines with theoretical and practical components, besides the suspension of activities in the field of practices, there was a need to reorganize theoretical content to maintain contact and interaction with students. The adoption of active methodologies was important to encourage this participation in the entire teaching-learning process and to promote the achievement of the subjects’ objectives.

In this context, the Project-Based Learning Methodology (PjBL) is highlighted, which allows students to confront real-world situations, issues, and problems, making them meaningful, determining how they should be addressed, and acting cooperatively in search of solutions. It is characterized as a methodology that encourages students to work collaboratively and in a team based way on the integration of different knowledge, stimulating the development of critical thinking and the active role of students, aiming to solve or propose the confrontation of a challenging issue stemming from the construction of a project. The PjBL methodology consists of the following steps: anchoring; driving question; investigation and research; creation and development; and presentation of results^(7)^.

The objective of this study was to report the experience of using the Project-Based Learning methodology in emergency remote teaching, with undergraduate nursing students.

## METHOD

This is an experience report on the use of the PjBL in the course of educational actions in nursing practice, developed remotely in 2020, during the pandemic caused by the new coronavirus, in the Bachelor’s and Licanciate Degrees in Nursing at a public university in the state of São Paulo.

The course has a workload of 90 hours, with a practical component of 60 hours, and is offered in the second semester of the first year of nursing school. It lasts for a semester, with a weekly meeting. The general objective of the course is *“to understand and apply the necessary foundations for the development of educational actions in health inherent to the practice of nursing in different social groups and learning scenarios”* ^(8)^. The objectives to be achieved by students are:

“1. To understand the concept of health education 2. To recognize the educational process as inherent to nursing practice 3. To identify and apply presuppositions of the theoretical frameworks of the learner’s adhesion and motivation in the educational practice. 4. To recognize and implement educational practice at different stages of life cycle. 5. Plan, build, develop, and evaluate educational health projects”^(8)^.

The course’s learning program, before the pandemic, adopted dialogic expository classes, case studies, workshops, discussions, and seminars as teaching strategies, with the support of a virtual learning environment, as well as practical activities (educational action in the field). The established assessment method included self and hetero-assessment, reporting, and activities developed in the field of practice. The approval criterion consisted of the final average above and/or equal to five and a frequency of at least 70%^(8)^.

The course plan remodeled from the PjBL methodology consisted of theoretical contents administered remotely in synchronous classes (expository and dialogic) through the use of the software of video calls *Google Meet* ^®^, through the corporate platform of the *Google Workspaces for Education* ^®^ in which students and professors are logged in the educational institution email. Asynchronous moments were supported by the institution’s virtual learning environment (VLE) *Moodle* ^®^.

The instructional design of the course’s VLE was structured following the contents covered and the phases of the PjBL, aiming to be a collaborative space for learning and exchanging experiences. The environment presented tools for asynchronous interaction between teacher and students; course guidelines and presentation; welcoming message from teachers to students; course schedule; notices and news board; course syllabus; division of tutoring groups; tasks, materials, and complementary bibliographies; assessment of classes and tutoring; as well as virtual classes and the recording of synchronous classes.

The evaluation method remained self- and hetero-evaluative, replacing the report and activities developed in the field of practices with the development and presentation of the educational project. At the end of the semester, students were invited to evaluate the course through a form on the platform *Google Forms* ^®^ composed of global assessment questions available in the VLE. For each proposition, the Likert scale was adopted, with four response intervals, ranging from one (totally agree) to four (totally disagree), as well as a space for students to add justifications to their answers.

### Project-Based Learning

At PjBL, students play an active role, being co-responsible for the construction of the project at all stages, seeking information, exchanging experiences, communicating and collaborating with their peers, in small groups, through the mediation of professors who act as tutors in the process^(7)^.

The methodology assumes the development of five phases: anchoring; driving question; investigation and research; creation and development; and presentation of results.

The phase **anchoring,** used to introduce the context of the project to students, can be presented in different ways, such as short narratives, data, and information from a certain context, as well as images, videos and/or news. The objective of this phase is to attract the students’ attention and interest, presenting the context and the problem to be worked on.

The phase **driving question** guides and identifies the focus of the context and of the problem that will be worked on in the project. It can be established in advance by the tutors or jointly identified by the students themselves, based on reflection, discussion, and the definition of priority questions related to the context and problem presented.

From the anchoring and driving question phases, students are encouraged to carry out the **investigation and research** for a better understanding and in-depth investigation of the problem, providing the articulation of theory with reality. The greater the engagement around the anchoring and driving question, the greater the involvement of students at this stage.

After investigation and research, students are encouraged to think, together and strategically, about the best way to create and develop forms of coping with the problem, characterizing the **creation and development** phase.

Finally, the group of students has the possibility to socialize the development of the project systematicaly in the **results presentation**, through diversified strategies, such as oral presentation, dialogue poster, video, among others. If the problem is related to a practical field activity, it can be shared with the subjects that make up the context worked on.

### Ethical Aspects

This experience report was built by the course professors and the creators of the project. The students were active participants in the execution of the pedagogical proposal. This research is part of Resolution No. 510 of April 2016, (Item VIII, Article 1) of the National Health Council (*CNS*), not requiring submission to the Human Research Ethics Committee^(9)^.

## RESULTS

The course planning took place at the end of the first semester of 2020, when the COVID-19 pandemic was already installed. The pedagogical meetings gathered the seven teachers involved to rethink the strategies used to implement the use of PjBL. The first measure adopted was the training of professors to use the methodology, through the indication of readings and moments of discussion mediated by professors with expertise on the theme.

Sixty-seven undergraduates from the second semester of the Bachelor and Licentiate Degrees in Nursing took the course.

The course plan was structured for the remote format with synchronous and asynchronous activities. The synchronous activities were organized into two categories: lectures and dialogues and round tables covering structural topics of the didactic content of the discipline and tutorial meetings. The asynchronous activities were carried out to support and/or complement the contents covered, as well as to provide more time for the construction of the projects. These moments had virtual classes and support material available on the *Moodle* ^®^.

To start using the PjBL, the students participated in a conceptual theoretical class, with the purpose of getting to know the teaching methodology, which was recorded and made available in the course VLE. Subsequently, the students were divided into seven tutorial groups, using the Learning Style Inventory, aiming at forming heterogeneous working groups^(10)^. The Inventory proposes the division of individuals into four groups, characterized by each person’s learning styles, in: diverging, assimilating, converging, and accommodating^(11)^.

The tutorials were developed following the methodology phases subdivided into:

**Anchor and driving question phases –** took place in two tutorial meetings. In the first meeting, there was the reception of the students by the tutor and the elaboration of the didactic contract. In remote teaching, the elaboration of the didactic contract is essential, since the professor is far from monitoring the student’s learning, which cannot become an obstacle. New strategies shall be used so that the monitoring is effective and the didactic contract rules are followed.

After the reception and the didactic contract, the anchoring and driving question phases were discussed. The students were able to decide on the driving question, with the support of the tutor, being stimulated with reflections and questions about the population’s health needs, considering the personal and the tutorial group’s experiences. The selection of the project theme could be broad, as long as it included an educational action. This decision was important to motivate the students regarding the project development.

**Investigation and research phase –** in the third tutoring meeting, the students set out for investigation and research. Tutors and students got together to plan this phase and carry out the research at an asynchronous time. Students were encouraged to search for bibliographies from databases, including a survey of scientific production on the defined topic. The research was also carried out based on the gray literature, based on documents and materials derived from public policies and/or made available by scientific societies and associations.

**Creation and development phase** – the fourth and fifth meetings were dedicated to the project creation and development. At that moment, the group chose the digital technologies to be used according to the educational objectives and target audience. Research was necessary regarding its operation and access, as well as the possible need for training for its use. The free access resources, with intuitive usability and/or those that the students know were privileged to facilitate the use during the proposed time.

**Results presentation phase –** in the sixth and seventh meetings, there was the presentation of the works within each tutoring group, and the mediation, consisting of questions and reflections, aimed at improving the projects. At this point, a curatorship with experts was considered for an analysis of the content produced, observing elements or points in disagreement with the evidence on the theme.

**Chart 1.**
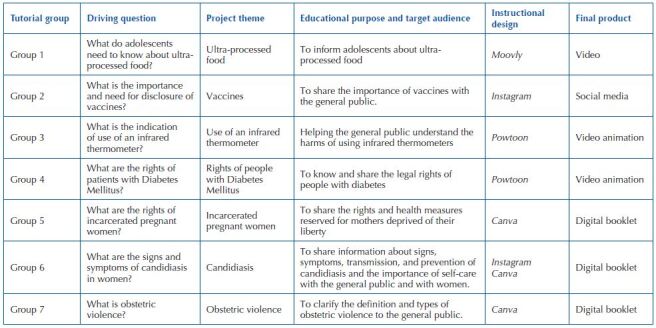
Themes of the projects presented by the tutorial groups

Subsequently, three asynchronous meetings were held to finalize the projects. In the last two meetings, totaling ten, projects were shared with all students and professors. At the end of the course, all projects were made available on the institution website. Project themes, educational purpose, Instructional design and final product presented below in chart 1.

**Figure 1.**
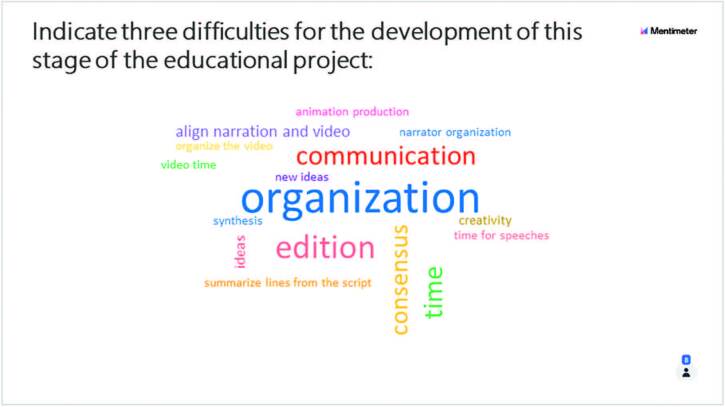
Difficulties raised at the end of the “Creation and development” phase of educational projects, based on the PjBL Methodology – Sao Paulo, SP, Brazil, 2020.

**Figure 2.**
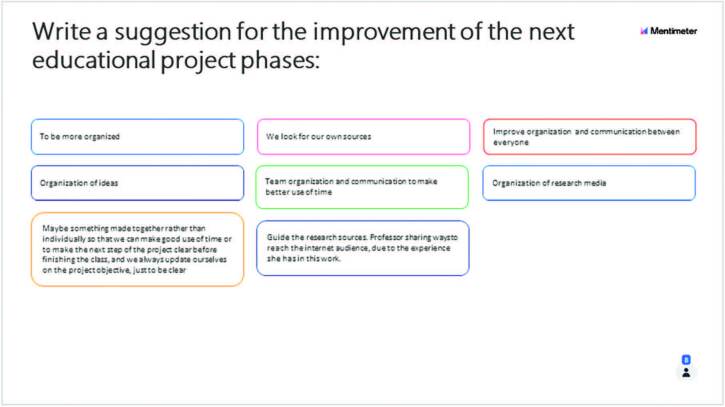
Suggestions for improvement to the difficulties encountered in the “Investigation and research” phase of educational projects, based on the PjBL Methodology – Sao Paulo, SP, Brazil, 2020.

Faced with the insertion of the PjBL in the course, there was a need to rethink the evaluation method, which became also procedural, through a formative evaluation carried out in each tutorial meeting, with the objective of placing the student and group performance within the teaching-learning process. The tutoring development evaluation was also carried out at the end of each phase of the PjBL, through the platform *Mentimeter* ^®^ with the following questions: 1. Indicate three difficulties for the development of this stage of the educational project (Figure 1); 2. Write a suggestion for the improvement of the next educational project phases (Figure 2). The figures represent examples of the evaluations of the “creation and development” and “investigation and research” phases of one of the tutoring groups:

The evaluation at the end of each phase of the PBLj was required to identify the difficulties encountered and define possible referrals based on the students’ perspectives and suggestions.

During the course planning and development, the interaction between students and professors involved dialogue, trust and respect for the differences between experiences, stimulating the satisfactory construction of knowledge. During the tutorings, the students developed working partnerships, proximity, progressing in the project construction and maturity.

With the *ERE,* some challenges emerged. The students started the nursing course already during the pandemic. Thus, they did not have the opportunity to meet each other in person, which had an impact on the integration and socialization among them and on teamwork. It was common to deal with students who did not turn the cameras on, because sometimes they did not want to, felt uncomfortable, or did not have the necessary equipment to do so. Difficulties with the internet and with the use of media equipment, both by professors and students, also affected the synchronous moments. Such difficulties faced by some students were discussed in the synchronous meetings, seeking the integration of them all. The issue was also dealt with institutionally, through the borrowing of electronic equipment and SIM cards for internet access.

Another challenge was the professor training on PjBL in a short period due to the pandemic context. Therefore, before the beginning of the course, a training period was planned, with prior reading about the methodology and discussion of the main points and phases of the PjBL. A presentation of the methodology to the students was also included, exploring the foundations and phases that would be used during the course. Both spaces were important to ensure the proximity and safety of teachers and students with the use of the methodology, with the performance of a professor with experience in PjBL operationalization being highlighted.

The final assessment carried out by the students through the *Google Forms* ^®^ demonstrates the effectiveness of the methodology used in relation to the achievement of the proposed objectives. The 67 students responded to the assessment. For 93.2% of the students, the objectives outlined for the course were achieved and for 97.3% the course contributed to the understanding of the role of nurses as educators; 90.4% agreed that the methodology adopted favored learning; 93.2% said that the stimulated autonomy favored learning, and 91.8% agreed that the course contributed to the development of reflective capacity.

## DISCUSSION

The course took place both asynchronously and synchronously, allowing students and professors to interact with the virtual learning environment to explore it to its full potential in the teaching-learning process. A study using an integrative literature review, which investigated the digital technologies used in nursing education for non-in-person teaching, such as virtual learning environment *Moodle*, *TeIEduc* and videoconferencing, teleconsulting, forum, website, software education, among other technologies, was highlighted^(12)^. Moodle was identified as one of the most used technologies for non in–person teaching. This environment allows the use of tools such as forum, chat, questionnaire, wiki-like texts, portfolio, among other possibilities, with flexibility in terms of the content to be taught. The authors emphasize the importance of professors looking for ways that provide interactive and innovative ways of teaching. In permanent education, the use of technologies in online training has also shown favorable results.

This integrative review analyzed skills and knowledge in the learning process of nursing students on the use of strategies for e-learning. It was identified that e-learning favors student self-assessment and measurement of clinical reasoning. The authors conclude that, although internet-based learning does not replace in-person learning, the combination of methods contributes to making it more effective. However, adequate training and preparation of the professor and student to use these resources are vital for the learning process success^(13)^.

Remote teaching is described in the literature as a collective challenge of building an educational scenario with geographically distant people, to create opportunities for interaction and promote individual and collective actions with the help of digital technologies. To this end, it is understood that the constitution of this scenario takes place in the sharing of experiences and in the improvement of professional understandings through collective actions between professors and students. Based on this perspective, the educational projects were built, founded on the PjBL methodology^(14)^.

The use of PjBL promotes the development of teamwork skills, encourages autonomy, proactivity, commitment, respect for the opinion of others, and the exercise of creativity. The association of active methodologies with Information and Communication Technologies (ICT) provides the development of the teaching-learning process^(14,15)^.

Learning emphasizes the protagonist role of the student, due to his/her direct, participatory, and reflective involvement in all stages of the process, experimenting, designing, creating, with the teacher’s guidance, the final product. Learning is developed in a flexible way through the sharing of space, time, activities, materials and technologies that make up the active process and project development^(15)^.

For some authors, *ERE* was presented concomitantly with limitations for the use of ICT in terms of cognitive, epistemological, and structural obstacles. The insecurities of teachers in the use of technologies, as well as cultural beliefs that students are more prepared for digital use, are pointed out as obstacles in adapting to the use of ICTs^(16)^.

In this context, there was a trend towards an increase in the number of hours dedicated to the use of ICT in 2020, with the most used resources being digital learning environments, followed by audio and video platforms. There were also limitations regarding the availability of equipment and the challenges of overcoming the inequality of opportunities among students, such as technological infrastructure and high-speed internet^(17)^.

ICTs are tools that allow new interactions and the creation of favorable spaces for the teaching-learning process, as they act as facilitators in the construction and exchange of knowledge, stimulating the exercise of autonomy of the subjects involved^(18)^.

Several authors highlight successful experiences in the educational process with the promotion of meaningful learning through PjBL, as learning is based on real problems, in the context of life, and the proposals for solutions are based on meeting these needs^(19,20,21)^.

In this regard, in the use of the PjBL methodology, the tutor’s supervisory role becomes essential, involving students in the creation and execution of the project, respecting the learning objectives and recognizing the difficulty that students and teachers have in planning and executing this strategy, which emphasizes the need for weekly records and monitoring to enable the construction of projects. The use of digital tools helps in the planning and execution of the project, as it promotes integration and agility^(21)^.

The PjBL methodology was very close to the subject’s learning object – related to the practical component, which was greatly affected by the pandemic –, allowing the student to develop an educational action in health. Based on the health needs identified by the groups of students, the methodology contributes to the construction of educational projects based on the current situation and its applicability.

In a study on pedagogical action in the light of PjBL, the path traced and the results obtained demonstrated the methodology as an effective strategy regarding the commitment to diversify the professor’s methodological repertoire, especially in a post-pandemic context that led to the establishment of a parallel, virtual world and to a reality of abrupt changes in the ways of interacting, dialoguing, teaching, and learning^(22)^.

At PjBL, students are protagonists and have a voice to make choices during the process. Thus, in this course, projects of educational actions were developed in different themes, supported by the theoretical methodological framework proposed during the execution of all projects^(7)^.

As a form of evaluation, the *Mentimeter* ^®^ was used, with the purpose and opportunity to re-signify the evaluation process with ICT as allies. The use of digital tools for the assessment process is effective and, when directed to formative assessment, allows students and professors to carry out agile, practical, remote, and synchronous feedbacks^(23)^.

## CONCLUSION

The ICTs are part of the contemporary model of teaching in health and nursing and tend to be more incorporated as scientific and technological development advances. In the course presented, the use of ICT was essential for the maintenance of the students’ academic schedule in the context of the pandemic, especially with regard to the practical component. However, the analysis and reflection on the limits and possibilities of the use of technologies in health and nursing education are required.

Offering the course with the use of PjBL, allied to technologies, proved to be effective in achieving learning objectives, especially in the development of skills to plan, build, develop, and evaluate educational health projects. The innovative character of the experience in articulating the PjBL with the promotion of the development of technological competences applied to health education in the training of nurses is also highlighted.

The tutoring meetings were fundamental for the growth and productivity of the group of students, allowing autonomy and freedom of creation and learning in topics related to health education, expressed by the satisfaction of students in the course evaluation.

Regarding the future challenges of the course’s professors, the main ones will be: the implementation of the methodology in the return of classroom teaching to support the performance and construction of projects to be applied in practice scenarios; and the reflection on the limits and possibilities of technologies for the development of technological competences in the training of nurses, aiming at their appropriate use in teaching, professional practice, and in the population’s health education.
